# The Clinical Teaching Fellow role: views of the Heads of Academy in the West Midlands

**DOI:** 10.1186/s12909-023-04219-y

**Published:** 2023-04-14

**Authors:** Isobel Marion Harris, Sheila Greenfield, Derek J Ward, Alice J Sitch, Jayne Parry

**Affiliations:** 1 Institute of Applied Health Research, University of Birmingham, Birmingham, UK; 2 NIHR Birmingham Biomedical Research Centre, University Hospitals Birmingham NHS Foundation Trust and University of Birmingham, Birmingham, UK

**Keywords:** Teaching fellows, Undergraduate medical education, Workforce, Teaching

## Abstract

**Background:**

Increasingly junior doctors are taking a year out of the traditional training pathway, and some opt to spend a year in a clinical teaching fellow (CTF) post. The CTF post mainly involves delivering hospital-based teaching to undergraduate medical students. In NHS hospital Trusts in the West Midlands, Heads of Academy (HoAs) have oversight of medical education at each Trust and therefore have responsibility for employing and directing the work of CTFs. Currently, only limited literature exists about the CTF role and exploring this from the point of view of different stakeholders in medical education is important in terms of contributing towards development of the role. This study aimed to explore the views of HoAs in the West Midlands region regarding CTFs employed at their Trusts.

**Methods:**

All HoAs at the NHS Trust/teaching hospitals associated with the University of Birmingham were invited to take part in an in-depth interview about CTFs at their Trusts. Interviews were held via Zoom recorded using Zoom’s recording functionality. Interview transcripts were then coded and analysed using thematic analysis.

**Results:**

Seven out of 11 HoAs participated in an interview. Seven themes were identified: CTF duties/Job role, Relationship with students, Benefits of having CTFs, Challenges associated with CTFs, Popularity of the role, What Trust offers CTFs, and Future of the role. Primarily it was felt that having CTFs at their Trust was beneficial in terms of the amount of teaching they provide for medical students. The HoAs were keen to ensure the CTF posts were of maximum benefit to both the post holders and to the Trusts where they were based. The CTF role is one that they felt would continue and develop in the future.

**Conclusion:**

This study has provided the first insight into the CTF role from the point of view of senior doctors with responsibility for delivery of undergraduate medical education. The consistency and reliability of teaching provided by the CTFs was identified as a key benefit of the role. Future work exploring the role from the point of view of post holders themselves would be beneficial to contribute to development of the role.

**Supplementary Information:**

The online version contains supplementary material available at 10.1186/s12909-023-04219-y.

## Introduction

In the UK, after graduating, junior doctors undertake two years of Foundation training before beginning a specialist training programme. Increasingly, junior doctors are now not proceeding directly into specialty training but opting to take a year out of training [1], commonly referred to as an “F3 year” [2]. One role that may be undertaken during an F3 year is the clinical teaching fellow (CTF) role. In the UK, CTFs are mostly employed on a one year contract and have responsibility for teaching undergraduate medical students in hospitals [3], although a small number of posts may be based at universities [4]. CTF job descriptions vary in the amount of time dedicated to clinical work alongside teaching responsibilities, and in terms of other duties within the role including research, student pastoral support, and obtaining postgraduate qualifications related to education [3, 5–7]. The CTF role has increased substantially in numbers in recent years – in 2005, there were 77 CTF posts in the UK [5], but as of 2018, one geographical area of the UK alone (North East England) reported 101 posts [6].

Currently, there is only limited literature available regarding the CTF role and the postholders themselves. Most of what exists is in the form of career advice or opinion pieces aimed at those who may be considering applying for a post themselves [3, 8–13]. A small number of additional studies have tried to map out what the role is [5], evaluate the CTF role from a undergraduate medical student point of view [14], and explored the views of CTFs and education faculty members based at a UK university [4]. The latter study explored the experiences of CTFs and university faculty members who had worked alongside each other, and specifically explored the views of the CTF position, as well as benefits and challenges associated with it. The study found that both CTFs and faculty members thought the role was beneficial in terms of career advancement and developing teaching-specific skills, and the role allowed post holders to have a better work-life balance through being outside of the main training pathway for a year. Some tensions were reported between the hospital and education faculty in terms of whether the CTFs should have a flexible or fixed teaching timetable. Whilst this study has provided an insight into the experiences of university-based staff members, there is currently no literature exploring the views and experiences of hospital-based staff working alongside CTFs which is where CTFs most commonly work.

The University of Birmingham College of Medical and Dental Sciences runs one of the largest Bachelor of Medicine and Surgery (MBChB) programmes in the UK, with up to 400 students in each of the five years of the course. At the University of Birmingham (UoB), the clinical teaching delivered to undergraduate medical students (that takes place externally at NHS hospital Trusts across the West Midlands region) is specifically managed through Clinical Teaching Academies (CTAs) which are each lead by a Head of Academy (HoA) under the direction of the University. CTAs are a formal grouping of the clinical and administrative staff involved in teaching and supporting medical students at each partner NHS Trust. HoAs are senior clinicians employed at a Trust who hold honorary contracts at UoB. The HoA is the main intermediary between the College of Medical and Dental Sciences at UoB and the NHS Trust in which the CTA is based. UoB had 11 HoAs with honorary contracts in 2020.

The HoA role associated with UoB has four areas of responsibility: leadership and management of education delivery and assessment, quality assurance, curriculum development, and student support. The main responsibility of leadership and management of education delivery and assessment involves managing the staff of the Academy and co-ordinating the work of clinical teachers to ensure the curriculum provided by the medical school is delivered and the assessments required as part of the programme are run effectively. As the HoAs manage the recruitment of teaching staff within their CTA, they therefore have responsibility for the CTFs, and are key in determining the duties of the posts.

This study aimed to explore the views of HoAs regarding CTFs using semi-structured interviews to give an insight into how such posts are utilised in a hospital setting and the experiences of staff members who have oversight for the posts. This could contribute to development of the CTF post to ensure it is of the most benefit to the post holders themselves, the medical students they teach, and the Trusts they are employed in.

## Methods

### Participants

All HoAs at each of the Trust/teaching hospitals associated with UoB and holding an honorary contract at UoB were invited to take part in the study (n = 11). An opportunistic sampling approach was taken where all eligible participants were invited to take part. This was appropriate for a study with a predefined population [15], and aimed to allow for saturation to occur which can be reached with relatively small sample sizes [16].

### Data collection

In July 2020, the study was introduced to the HoAs at their quarterly meeting with the College of Medical and Dental Sciences at UoB. Individual emails were then sent to the HoAs inviting them to participate in an interview at a time that was convenient to each person. Interviews took place between September and December 2020 and were held virtually via Zoom [17]. The interviews explored the participants’ experiences of CTFs at their Trusts (see appendix 1 for topic guide) by asking what the CTF role looked like at their Trust, what the benefits and challenges of having CTFs were, what the relationship between CTFs and students was like, and about how they thought the role might be used and develop in the future. The interviews lasted between 40 and 75 min, and were recorded using Zoom’s recording and automatic transcription functionalities. All participants were interviewed by IH, a female in her thirties, who is a doctoral researcher and research fellow in Applied Health Research at UoB. IH had previously been registered as an undergraduate medical student at UoB approximately 10 years prior to this study and had had clinical placements at three of the Trusts that participants in this study were employed at. IH introduced herself as a doctoral researcher, but unless specifically asked, did not say she had previously been a medical student to avoid this having any influence on the interview.

### Data analysis

The interviews transcripts were analysed using thematic analysis [18]. This involved coding data and identifying themes in the data. As this study was exploratory in nature, an inductive approach was taken for the analysis [19]. This meant the analysis was not approached with any particular theories or preconceived ideas about what the data may contain, but was instead led by what was in the data [19]. The interview transcripts were uploaded into NVivo 12 Plus [20] where initial coding took place. The codes were then exported into an *Excel* spreadsheet where related codes were arranged into candidate themes. These candidate themes were then reviewed by reading the coded extracts that had been sorted into each theme. A decision was made as to whether they fitted within that theme and were suitably related to the other extracts housed within that theme or if they were better suited elsewhere. The transcripts were then reread to assess whether the themes generated were reflective of the dataset. All data was coded by IH and regular meetings were held with other researchers all of whom have involvement with medical education (JP, Professor of Public Health, female, SG, Professor of Medical Sociology, female, DW, Reader in Public Health and Medical Education, male, AS, Senior Lecturer in Biostatistics, female) to discuss and refine coding and analysis.

### Ethical considerations

Ethical approval was granted by UoB in May 2020 (ERN_19-0687B).

## Results

Seven of the 11 HoAs from the 11 Trusts that provide medical student placements for UoB Medical School agreed to take part in an interview, two declined (one because they did not have CTFs at their Trust, and the other because they had resigned from the HoA role), and two did not respond to the invitation (response rate 64%). The seven Trusts that the participating HoAs were based at are not named in this paper to ensure confidentiality for the participants. The Trusts varied in terms of location within the West Midlands region, size, whether they taught students from one or two different universities, and whether they offered general or specialty only services (e.g. maternity, psychiatric, paediatric). Six of the seven Trusts represented currently had CTFs and one was planning to recruit the first CTFs in the coming year. Table 1 describes the characteristics of the Trusts at the time of interview.

**Table 1:** Characteristics of participating Trusts

Trust	General/Specialty	Number of universities	CTFs
1	General	1	Y
2	General	1	Y
3	General	2	Y
4	General	1	Y
5	General	1*	Y
6	Specialty	1**	N
7	Specialty	1	Y

*In negotiations with two other universities

**To take students from second university in October 2021

**Fig. 1:**
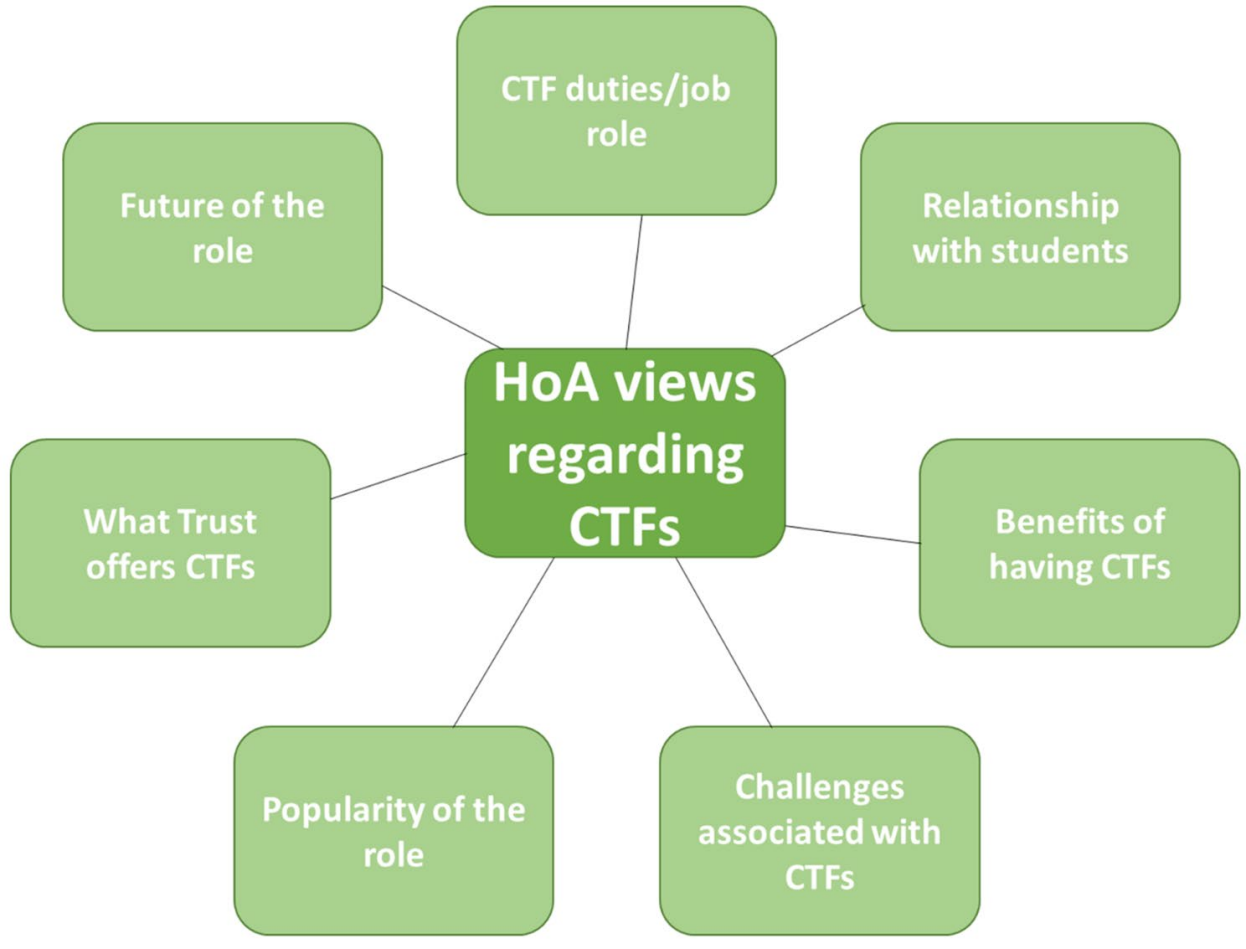
Themes identified from the interviews

Seven major themes were identified from the data which turned out to reflect the topic guide: CTF duties/Job role, Relationship with students, Benefits of having CTFs, Challenges associated with CTFs, Popularity of the role, What Trust offers CTFs, and Future of the role (Fig. 1).

### CTF duties/Job role

All the HoAs described the same basic duties for their CTFs - providing teaching to medical students, and planning/co-ordinating that teaching. However, there was variation across the Trusts in terms of other aspects of the job. For example, at one Trust, the role was described as being 100% teaching, whereas at other Trusts the role also involved some clinical work alongside teaching. This ranged from a 50/50 to an 80/20 split teaching/clinical role. Other variations included the role being reported to include simulation teaching, teaching to non-medical students/staff, and providing mentoring for students. One Trust reported having additional educational fellow posts that were filled by healthcare professionals other than doctors e.g. a pharmacist and a nutritionist.

The minimum level of experience several HoAs reported considering for someone applying to a CTF post was completion of the first two years of Foundation Training. Several HoAs expressed the desire to hire as senior a person as possible to the role, with reasons for this including senior trainees having more clinical experience, broader knowledge, and more confidence in teaching. Despite expressing the desire for seniority in the role, it seemed that most posts were filled by doctors at a more junior career stage, and this appeared to be whom the majority of applications were currently coming from. Participants described how they had noticed a shift toward more junior doctors being interested in the CTF role, and offered reduced opportunities to take time out or pause training in specialist training posts as a suggestion for why they thought more junior doctors are applying for the CTF posts.

“*Initially, we were all most probably keen to have some senior guys like the people who wanted to, you know, specialist training and we did have the guy who was at the end of their surgical training, of course, they are the senior guys that most probably have better knowledge and they do teach differently. But the dynamics recently, I’ve seen like last few years have changed, the lot of local doctors take a year out, you know, after FY2 and they would like to do the teaching job*.” (Participant 4).

Another offered reduced flexibility in specialist training posts as a suggestion for why they thought more junior doctors are applying for the CTF posts.

“*We’re keen to recruit doctors as seniors as we can, but often we will recruit doctors at the completion of the F2 year…When I was teaching fellow in 2008 I was a third or fourth year specialist trainee. And the teaching fellows I worked with were all specialist trainees, so this is an example of how the role as the flexibility of specialist training or what were called SPR posts was reduced and therefore it’s more difficult to step out as an SPR to teach than it was previously*.” (Participant 1).

### Relationship with students

All of the HoAs that had CTFs at their Trust reported that students have good relationships with CTFs, and both like and appreciate them. Reasons for this included the CTFs being seen as being approachable, the students being able to relate to the CTFs as they have recently been medical students themselves, and the students finding them less intimidating than more senior staff.

“*What is abundantly clear is that, how much the students really, really appreciate the teaching fellows. And I think they appreciate them much more than even the trainees who are within their specialty areas. I think the teaching fellows form quite a special bond with the students. And I think the students feel very comfortable at exposing their own ignorance to the teaching fellow, which they won’t do to more senior people perhaps*.” (Participant 2).

Participants also thought that the closeness in age between students and CTFs was an important factor in the good relationship between the two.

“*I think there is a value in students being closer in age, maybe also, they may be engaged more easily*.” (Participant 7).

### Benefits of having CTFs

All of the interviewees felt that it was beneficial having CTFs at their Trust, or in the case of the Trust planning to recruit CTFs, that it would be beneficial to have CTFs. A view shared by several participants was that CTFs are beneficial in terms of the amount of time they are able to dedicate to teaching medical students compared to other clinicians.

*“Well, I mean, I think, well, they’re everything really, they’re invaluable but in terms of just having six full time people to teach, what’s that, that’s 240 hours a week essentially of time that, there’s no way you’d ever get that many… and I don’t know how we managed before we had them actually.”* (Participant 3).

Being a reliable teaching resource was seen as being key to consistently providing enough teaching for the medical students, something that other clinicians struggle to do with other responsibilities to fulfil.

“*Because we’re a small Trust, we have a limited number of consultants and they’re all on massive job plans, and I think without the fellows, we would struggle to provide enough education*.” (Participant 5).

“*The teacher fellows have a more stable and less chaotic job plan and day to day activity. So it means they can be relied upon to be where you’d like them to be more so sometimes than clinicians who have other roles*.” (Participant 1).

Other benefits of having CTFs were identified as the CTFs having innovative ideas on how to deliver teaching, and that they are knowledgeable about what students do and do not know, with the implication being that they can tailor their teaching appropriately to this.

*“I think a lot of time they [CTFs] come with ideas, you know, and it’s good to run with them because a lot of the time they have fresh ideas of how things should be taught. Usually much better than us oldies on how to deliver things virtually or use technology etc.”* (Participant 6).

### Challenges associated with CTFs

The challenges associated with having CTFs were split broadly into two categories; those to do with the work environment, and those to do with the CTFs themselves.

With regards to the work environment, HoAs identified issues with IT impacting upon the CTFs (e.g. log ins not working across multiple sites), and not having sufficient space within the hospital for the CTFs to work in (e.g. not having enough dedicated office space).

When talking about challenges with the CTFs themselves, HoAs gave the impression that challenges were minor, usually rare occurrences, and that the benefits of having CTFs far outweighed anything else. Examples given suggested that issues were related to the job itself being very different from usual clinical work, and requiring different skills and different ways of working.

“*[Planning of teaching programmes is] something that’s new, that they wouldn’t ever done before and something they need to learn, it’s a new skill and it can be challenging because they’ll not be used to sitting in offices, working with coordinators and non-medics and that sort of stuff*.” (Participant 3).

Sometimes this could result in problems with engagement or motivation. Some HoAs also described isolated incidents where they had had someone in a CTF post who did not meet their expectations.

“*Very rarely do I get fellows who doesn’t perform, and last year, I had one and then I had another one about five years before that. Part of the difficulty was he wasn’t giving the time to the sessions that I wanted him to give and I didn’t know where he was when he wasn’t teaching.*” (Participant 5).

### Popularity of the role

The HoAs who had CTFs at their hospital reported that they did not struggle to fill the posts each year and had more applicants applying than they had places. HoAs offered the views that the role was popular and liked by CTFs because it was lifestyle-friendly with more predictable hours and less anti-social shift patterns, it offered an opportunity to do something different, it offered a break from the normal training pathway, and it offered a great deal in the way of development opportunities both in terms of medical education and other interests.

“*Lots of people don’t want to go straight from foundation into a training program. They want a year to think about things and see what they like, what they don’t like. Some people take it for lifestyle reasons. Don’t want to do on calls for a year for example. Some of the more senior trainees have had a moment in life thinking, am I in the right specialty, and wanted to step off and take a moment to think.*” (Participant 2).

Some HoAs attributed the popularity of the CTF post to how having the opportunity to gain a qualification and having the time for other experiences that may not be possible in a normal clinical role could help to give an advantage when applying for future jobs.

*“There may be a little bit of more competition now to go to different speciality, and if they have done an educational certificate, and they are being given, you know, both probably something towards their interviews, you know, which helps them. And I also feel there’s a broad expectation of these role nationally. When the candidate has done like a dedicated teaching year with some postgraduate certificate, a diploma, has presented maybe a couple of papers, it certainly carries a lot of weight in the interviews for either if this is a CT job or ST job*.” (Participant 4).

### What the Trust offers CTFs

It was very clear from the HoAs that the CTFs provide great benefit to the teaching life of the Trusts, and it was also evident that the HoAs were very keen for their individual Trusts to offer benefit to the CTFs themselves. Some HoAs described specific elements in the job role that would be beneficial to the post holder themselves, and also beneficial in attracting the best candidate to the post. These included things like dedicated study time, being allowed to pursue own clinical interests, and developing skills outside of medical education. There was an awareness from the HoAs that whilst there was no shortage of candidates applying to the posts, their Trust needed to ‘stand out’ to attract the most desirable candidates. This was in synergy with wanting to offer worthwhile development opportunities to the CTFs.

“*So what I did was, to get the best people, I allowed a nominal, a session a week of what they wanted to do clinically, because I was aware that if you ask the trainees to take an out of programme year, you want the best people. And so you’ve got to make it attractive and some of them might actually not really be motivated completely about teaching, but they are excellent teachers because they’re talented, you know? So I don’t mind having someone who main interest is that they want to learn [particularly clinical area].*” (Participant 7).

*“I encourage the CTFs, my main goal is to encourage them to try and expand their horizons as wide as possible and champion things outside of teaching. So what can they take into writing, publication or those kinds of things, which is really important.”* (Participant 1).

### Future of the role

All of the HoAs saw the CTF role as persisting into the future and had various ideas about how the role might develop. Several mentioned the idea of having or expanding the number of non-medic CTFs which they felt would be beneficial in providing broader teaching e.g. recruiting physiotherapists/other allied health professionals, and others had ideas such as specialty specific CTFs to help provide consistent teaching in busy areas.

“*For example, [the emergency department] which is always a pressure cooker, it’s really, really busy all the time, and sometimes clinicians don’t have the time to give to the students as much as they would like. And again, if we had a dedicated ring-fenced A&E teaching fellow, for example, then, then when they come to that area, even if the place is heavingly busy, the students will still get the attention that they deserve*.” (Participant 2).

“*The money that I haven’t used to fill those medical clinical education posts, I’ve used to develop an education fellow posts within the allied health professionals… For me, it’s a way of giving our medical students a little bit…just a bit of diversity really, you know, I think it’s really important for the medical students that they realise that they aren’t just working with doctors, they’re working with other healthcare professionals and it just kind of gives… I mean, they’ve learned so much. I mean, the feedback from the pharmacy sessions are just, it’s just been phenomenal really and so I’m quite excited about that*.” (Participant 5).

Some talked about downstream benefits of continuing to have CTFs such as flexible career paths developing that involve specific time allocated for education, and the end result of having more educationalists in senior positions.

“*I think this role is going to develop more and more, and every Academy and every organisation will probably look at differently, you know, to how they how they employ these doctors and may well engage them part time, you know, teaching part time and working part time in a dedicated specialty. There might be some more roles coming out of this like leadership and management roles. But what I’m seeing is this is a more educational role. Most probably, we will see more educationalists who will develop into being a consultant and they will look at some more dedicated educational jobs*.” (Participant 4).

## Discussion

To best of the authors’ knowledge, this is the first study to explore the views of senior hospital doctors with responsibility for education regarding the role of CTFs at their Trusts. In depth interviews conducted with seven HoAs in the West Midlands region have provided insight into a previously unresearched area with no directly comparable existing literature, and shown how CTFs fit into the educational life of Trusts included in this study. The topic guide for this study asked broad questions to address our aim of exploring the views of HoAs regarding CTFs and resulted in a broad range of discussion being surfaced. The themes identified mostly reflected the topic guide with a couple of additional themes found.

It is clear that for the HoAs interviewed, having CTFs at their Trust was felt to be beneficial in terms of the amount of teaching they provide for medical students, and that the CTF role is one that they will continue within their Trust, and may develop in terms of who would be hired to that post, or the specific teaching they would provide.

To a certain degree, it is not surprising that the HoAs described variations in the duties of the CTFs at their individual hospitals. CTF posts are outside of the usual training pathway, and there is no national standard for what the job should entail, therefore meaning that each Trust is responsible for hiring and determining the responsibilities of the posts themselves. Whilst the basic teaching duties at each Trust were similarly described, variations in duties could mean that posts with the same title actually end up being very different jobs. In this study, the Trusts that the HoAs were from primarily received students from one established medical school, with some also receiving the first year of clinical students from a new medical school. Broadly, this means that all the Trusts were delivering the same curriculum, yet still the posts hired to deliver this teaching showed significant variations. It would be interesting to further explore why different Trusts utilised the CTF role in different ways, and to know at a national level how much CTF jobs vary.

For the majority of the areas discussed in the interviews, the HoAs were mostly all in agreement, giving similar answers and opinions, particularly regarding the benefits of having CTFs, and aspects of the role such as popularity of the job and relationship with students. There are several possibilities as to why this could be, for example, there was a small number of participants in this study, and they all worked in similar roles. It would appear that the CTF role partly exists to solve the problem of clinicians not having enough time to teach as found in previous research [21]. With increasing numbers of medical students and increasing clinical pressures in hospitals, it is therefore not surprising that all participants viewed the CTFs in a positive light as the CTFs seem to provide a convenient and reliable solution to these pressures. Diverging views may have been obtained had other clinicians been included in this study, for example, those without any special interest or role in medical education.

CTFs seem to provide similar benefits to that provided by near-peer teaching, particularly in terms of reducing teaching burden on other members of staff [22] and to a degree, sharing cognitive and social congruence with medical students [23]. Participants in this study identified CTFs being closer in age to medical students, and being seen as less intimidating than more senior members of staff as reasons why the students and CTFs had a good relationship. Whilst this shows similarities with near-peer/peer assisted learning, CTFs are however fulfilling a different role as they are further removed from the medical students in terms of career-stage than near-peer teachers are, and CTFs are a dedicated teaching provision role rather than being students themselves. The CTF role could be seen to be as an extension and formalisation of near-peer teaching.

It was clear from most of the participants that the number of CTFs at each Trust had been steadily increasing since they first began to have CTFs, and several indicated they were either already planning to recruit more or were trying to ringfence money to recruit more in the future. As mentioned above, part of this continued increase in popularity for having CTFs could be driven by Trusts seeing what other local Trusts are doing and basing their decisions on that. All the HoAs reported that CTFs were providing a solution to an increased teaching demand that was beneficial to both the hospital and the students, but it is however a relatively new and unassessed role.

There was an awareness from the HoAs that although the CTF posts are popular, with some interviewees reporting they had more applicants than places, the posts offered by the Trusts need to be competitive with the benefits they offer the post holders. The HoAs indicated reasons other than a desire to teach for why CTFs opted for the role such as wanting a more lifestyle friendly job, and wanting something to help with future competitive specialty applications. It seemed that the posts offered by the HoAs interviewed had been designed with this in mind, for example, by including sessions of clinical work as decided by the post holder and encouragement of wider academic activities. This therefore means the CTF roles are beneficial to the Trust in terms of providing teaching solutions, and also beneficial to the post holders in terms of being tailored to personal development needs and desires. It would be interesting to explore CTFs motivations for choosing a teaching fellow post and understanding what features make posts particularly attractive.

Of additional interest is the idea of offering benefits to post holders to attract the ‘best’ candidates. These interviews did not explore what makes the ‘best’ CTF, and when describing what they liked about having CTFs at their Trusts, the HoAs listed good relationships with students, having the time to provide teaching, and bringing innovative ideas as key points. No specific mention was made of teaching ability or qualifications ahead of the role, although when describing why more senior trainees may be preferrable, wider knowledge and more teaching experience was mentioned. Future work to explore the attributes of ‘good’ CTFs and the importance of teaching ability is warranted.

An additional area that would benefit from future research is the idea of education as a career option for doctors. The HoAs thought that CTFs may go on to have careers involving education in some way or develop into senior educationalists, but it is not known whether those who have held a CTF post would be interested in such a career or if the opportunities/infrastructure for these sorts of jobs is in existence. In depth work with CTF post holders exploring this idea would be useful.

### Strengths and limitations

This is the first study of its kind carried out with senior doctors in a HoA role. In depth interviews have provided novel insights into a key area of undergraduate medical education from the point of view of HoAs, and identified some areas for future research.

This study does have several limitations however. Firstly, participants were from one region, the West Midlands, therefore the results from this study may not be transferable to other areas. Whilst undergraduate medical education is broadly managed in the same way across the country [24], there may be differences in the organisation and delivery of education that would mean that education leaders have different experiences to those included in this study.

Secondly, not all invited participants took part, and it may have been that those who declined had differing views and opinions, and the characteristics of their Trusts were not gathered as part of this study. However, participants did come from Trusts that varied in terms of year groups taught, whether they were a specialty or general Trust, and whether they currently had CTFs or not, so it is likely they reflected the range of Trust characteristics.

Thirdly, only a small number of people hold a HoA role in the West Midlands which limited the number of potential participants, and could have impacted upon reaching data saturation. Ideally, there would have been a large number of potential participants that could have been invited to take part in the study, and interviews would have been carried out until reaching data saturation, where no new data was being generated [25]. However, the HoAs interviewed for this study did tend to have similar opinions on all of the topics covered and therefore, despite the small number of participants, it is felt data saturation was reached.

## Conclusion

The CTF role is still a relatively new role, and as shown in this study, one that has not yet been adopted by all Trusts in the West Midlands. There is currently no directly relevant literature available exploring this topic, and this study has provided the first insight into the views of HoAs about the role. With HoAs ultimately responsible for the co-ordination and delivery of undergraduate medical education, and therefore the duties of the CTFs, at their Trusts, it is beneficial to explore their views regarding the role to gain an understanding of the perceived benefits and challenges of the role. With Trusts able to make individual decisions about the use of their CTFs, this study adds value by comparing views and practices across several Trusts. This research will be of interest and use to anyone who has or is considering employing CTFs (or equivalent roles e.g. other allied health professionals) within their own clinical environment.

Exploring the CTF role from the perspective of HoAs with the descriptions of difficulties and challenges faced by HoAs in terms of ensuring teaching for undergraduate medical students has helped to provide some context for why they perceive the CTF role as being beneficial and useful to the Trust. CTFs being able to provide a reliable source of teaching is particularly beneficial meaning HoAs know there is consistency in the teaching provided to medical students at their Trusts. The relative newness of the post, combined with a lack of literature, clearly indicates that future research exploring how the CTF role continues to be utilised and develops is merited. Of particular interest would be more exploration of the views of CTFs themselves to gain a fuller understanding of the post from the perspective of the post holders, helping to contribute to the development of the role in to one that is of maximum value to post holders, medical students, and hospital Trusts.

## Electronic supplementary material

Below is the link to the electronic supplementary material.

Supplementary Material 1

## Data Availability

The datasets generated and/or analysed during the current study are not publicly available due to participant confidentiality but are available from the corresponding author on reasonable request.
