# Supplementary material for: The Clinical Teaching Fellow role: views of the Heads of Academy in the West Midlands
Source: BMC Med Educ. 2023 Apr 14;23:242. doi: 10.1186/s12909-023-04219-y (PMC10103451; doi:10.1186/s12909-023-04219-y)
Supplement: Supplementary file 1 — Supplementary Material 1 [file 12909_2023_4219_MOESM1_ESM.docx]

**Appendix 1**

**Heads of Academy interview topic guide**

- Tell me about CTFs at your trust – when did you first have them, what are their responsibilities, how has the role changed
- Why do you have them?
- What works well?
- What doesn’t?
- How do you think the students feel about CTFs?
- How do you see the role being used in the future?
